# Epidemiology of Obese Patients Undergoing Revision Total Knee Arthroplasty: Understanding Demographics, Comorbidities, and Propensity Weighted Analysis of Inpatient Outcomes

**DOI:** 10.5435/JAAOSGlobal-D-21-00263

**Published:** 2022-02-16

**Authors:** Inaya Hajj Hussein, Abdul Kareem Zalikha, Andrei Tuluca, Zachary Crespi, Mouhanad M. El-Othmani

**Affiliations:** From the Department of Biomedical Sciences, Oakland University William Beaumont School of Medicine, Auburn Hills, MI (Dr. Hussein); the Department of Orthopaedic Surgery and Sports Medicine, (Dr. Zalikha, Dr. El-Othmani), Detroit Medical Center, Detroit, MI (Dr. Zalikha and Dr. El-Othmani); and the Central Michigan University College of Medicine, Saginaw, MI (Tuluca, Crespi).

## Abstract

Obesity is a public health epidemic that is projected to grow in coming years. Observational data on the epidemiologic profile and immediate postoperative outcomes of obesity and morbid obesity after revision total knee arthroplasty (rTKA) are limited.

**Methods:**

Discharge data from the National Inpatient Sample was used to identify patients who underwent rTKA from 2006 to 2015. Patients were stratified into morbidly obese, obese, and not obese control cohorts. An analysis was performed to compare etiology of revision, demographic and medical comorbidity profiles, and immediate in-hospital economic and complication outcomes after rTKA.

**Results:**

An estimated 605,603 rTKAs were included in this analysis. Morbidly obese and obese patients were at significantly higher risk for any complication than not obese patients. Patients with obesity were associated with an increased risk of postoperative anemia but a lower risk of peripheral vascular disease and gastrointestinal, and hematoma/seroma complications compared with not obese patients. Patients with morbid obesity were associated with an increased risk of any, hematoma/seroma, wound dehiscence, postoperative infection, pulmonary embolism, and postoperative anemia complications and a lower risk of gastrointestinal complications when compared with not obese patients. Morbidly obese patients had a significantly longer length of stay than both obese and not obese patients, while no significant difference in length of stay was observed between obese and not obese patients.

**Discussion:**

Morbidly obese patients are at higher odds for worse postoperative medical and economic outcomes compared with those with obesity after rTKA. As the number of patients with obesity and morbid obesity continues to rise, these risk factors should be considered in preoperative discussions and perioperative protocol optimization.

Primary total knee arthroplasty is a successful procedure for end-stage arthritis in relieving pain and regaining function. However, revision procedures such as revision total knee arthroplasty (rTKA), for reasons such as mechanical loosening, stiffness, and infection, are associated with notable patient morbidity and financial burden to the healthcare system.^1,2^ This financial burden has been growing in parallel with the steadily increasing rates of rTKA, which is projected to increase between 78% and 182% from 2014 to 2030.^3^ Given that rTKA is associated with notable complications and worse functional outcomes than primary procedures,^4,5,6,7^ the demographic and comorbidity profiles of patients undergoing these procedures need to be understood, and hence patient-specific risk factors for postoperative complications be identified, which may better guide perioperative optimization efforts.

Obesity is a public health epidemic that continues to burgeon globally, with recent analyses projecting nearly half of adults will be obese and 24.2% will be severely obese by 2030.^8,9^ Although obesity has been proven as a risk factor of poor outcomes after primary TKA,^10,11,12,13^ literature remains lacking regarding the assessment of the demographic and medical comorbidity profile of obese patients undergoing rTKA and the effect of those factors on immediate in-hospital postoperative outcomes. An early study by Bieger et al reported that body mass index (BMI) had no effect on complications after rTKA, whereas other studies noted a correlation between obesity and complications, readmissions, and worse functional outcomes after rTKA.^14,15,16,17^ In addition, the potentially different influence of morbid obesity when compared with obesity on postoperative outcomes is little understood. Overall, a better understanding of this patient population characteristics and the effect of obesity and morbid obesity on postoperative complications and economic outcomes after rTKA is important for perioperative optimization and patient counseling and education, especially given the growing subset of obese patients among rTKA recipients.^18^

Within this context, the purpose of this study was (1) to evaluate the trends in type and etiology of rTKA among obese patients, (2) analyze the differences in demographic and medical comorbidity profiles between obese, morbidly obese, and control groups, and (3) compare immediate in-hospital medical and economic outcomes between these three groups.

## Methods

A retrospective analysis of discharge data from 2006 to the third quarter of 2015 using the National Inpatient Sample (NIS) registry, which is part of the Hospital Cost and Utilization Project, was performed. This database incorporates and accounts for approximately 20% of inpatient stays within the United States.^19^ The NIS database includes information such as patient demographics, charges, comorbidities, and perioperative outcomes. The *International Classification of Disease, Ninth Revision, Clinical Modification* (ICD-9-CM) was used for procedure/diagnosis codes within the NIS during the study period. Because the NIS transitioned to ICD-10 coding in the fourth quarter of 2015 and to maintain homogeneity of methodology and included database, we elected to exclude that quarter.

Patients aged at least 40 years who underwent rTKA were included in this study. These patients were identified with ICD code 81.55 (revision of TKA). Reasons for revision were accounted for using the following ICD codes: infection (996.66), mechanical loosening (996.41), other mechanical problems (996.47), implant failure (996.43), dislocation/instability (996.42), other mechanical complications (996.49), bearing surface wear (996.46), periprosthetic osteolysis (996.45), and periprosthetic fracture (996.44). The type of revision was also reported: all implants (00.80), arthrotomy for removal of prosthesis (80.06), other, not otherwise specified (81.55), tibial insert (00.84), tibial implant (00.81), patellar (00.83), and femoral implant (00.82).

Discharge weights, clusters, and strata were all accounted for as recommended by the Agency for Healthcare Research and Quality. Once rTKA patients were identified, they were stratified into one of three groups: obese BMI between 30 and 40 kg/m^2^ (ICD-9-CM 278.00), morbidly obese BMI > 40 kg/m^2^ (ICD-9-CM 278.01), and not obese as a control cohort.

Inverse probability of treatment weighting, which is a validated method of balancing covariates that helps minimize the effect of confounding bias, was performed.^20^ Propensity score weighting was performed using the method described by DuGoff et al^21^ by weighting patient demographics, hospital characteristics, and comorbidities using the Elixhauser comorbidity index. By incorporating multiple different comorbidities, the Elixhauser comorbidity index is commonly used in large database research to properly assess patient comorbidities. It is superior to other comorbidity indices in controlling for potential confounding effects of preexisting diseases.^22^ We thus elected to use the inverse probability of treatment weighting/propensity score weighting incorporating the Elixhauser comorbidities, in addition to patient demographics and hospital characteristics, as the statistical method to control subject for confounding effects. Given the three cohorts, this approach allowed for controlling for comorbidities without losing a large number of patients that would occur with standard matching methodologies and was as such successful in the setting of large epidemiological studies like this one.

The variable any complication was used as a composite measure referring to any cardiac, respiratory, peripheral vascular disease (PVD), hematoma/seroma, wound dehiscence, postoperative infection gastrointestinal (GI), genitourinary, deep vein thrombosis, pulmonary embolism, or postoperative anemia in-hospital complication. Patient demographics and comorbidity profiles, in addition to immediate in-hospital length of stay, disposition, and economic outcomes, were comparatively analyzed using these weighted cohorts. Continuous and categorical data were analyzed using Student *t*-test and univariate logistic regressions, respectively. Statistical significance of the data was defined at a *P* value < 0.05. All statistical analyses were performed using SAS 9.4 (SAS Institute) for Windows.

## Results

### Demographic Data and Medical Comorbidities

A total of 605,603 rTKAs were included in this study. Based on the data reviewed from the NIS database, the relative proportion of patients with obesity (6.36% versus 11.04%; *P* < 0.0001) or morbid obesity (3.4% versus 12.57%; *P* < 0.0001) increased from 2006 to 2015. A statistically significant difference was observed between control, obese, and morbidly obese groups in which morbidly obese patients were more likely to be younger (62.17 years versus 64.28 for obese versus 66.56 for control subjects, *P* < 0.0001), female (68.42% versus 60.6% for obese versus 55.44% for control subjects, *P* < 0.0001), of Black race (13.21% versus 10.86% for obese versus 8.25% for control subjects, *P* < 0.0001), using Medicaid and private insurers as primary payors (5.87% versus 4.21% for obese versus 3.84% for control subjects, and 35.56% versus 34.24% for obese, versus 30.25% for control subjects, respectively, *P* < 0.0001). Interestingly, morbidly obese patients were more likely to receive their revision procedure under a nonelective admission, an observation not mirrored in the obese cohort (21.54% versus 16.59% for obese versus 17.37% for control subjects, *P* < 0.0001). The detailed demographic variables of the study population are summarized in Table 1.

**Table 1:** Demographic and Hospital Factors, Stratified by Morbid Obesity, Obesity, and Control Groups

Factor	Not Obese (n = 465,968)	Obese (n = 70,953)	Morbidly Obese (n = 68,682)	*P*
Age of patient (yr)				
Mean (standard error)	66.56 (0.06)	64.28 (0.09)	62.17 (0.09)	<0.0001
Elective admission				
Nonelective	80,947 (17.37%)	11,773 (16.59%)	14,791 (21.54%)	<0.0001
Elective	383,949 (82.40%)	59,003 (83.16%)	53,756 (78.27%)	
Unknown	1,072 (0.23%)	177 (0.25%)	136 (0.20%)	
Biological sex of patients				
Male	207,630 (44.56%)	27,959 (39.40%)	21,688 (31.58%)	<0.0001
Female	258,338 (55.44%)	42,994 (60.60%)	46,995 (68.42%)	
Primary payor				
Medicare	279,009 (59.88%)	39,011 (54.98%)	36,641 (53.35%)	<0.0001
Medicaid	17,903 (3.84%)	2,986 (4.21%)	4,032 (5.87%)	
Private	140,970 (30.25%)	24,297 (34.24%)	24,420 (35.56%)	
Self-pay	2,474 (0.53%)	332 (0.47%)	247 (0.36%)	
Other	25,611 (5.5%)	4,327 (6.1%)	3,342 (4.86%)	
Race of patient				
White	330,671 (70.96%)	48,813 (68.80%)	47,427 (69.05%)	<0.0001
Black	38,451 (8.25%)	7,703 (10.86%)	9,075 (13.21%)	
Hispanic	19,462 (4.18%)	3,473 (4.90%)	2,751 (4.01%)	
Asian or Pacific Islander	3,413 (0.73%)	318 (0.45%)	266 (0.39%)	
Native American	1,978 (0.42%)	288 (0.41%)	330 (0.48%)	
Other or unknown	71,991 (15.45%)	10,357 (14.60%)	8,834 (12.86%)	
Year of discharge				
2006	39,303 (8.44%)	4,510 (6.36%)	2,336 (3.40%)	<0.0001
2007	42,324 (9.08%)	4,914 (6.93%)	2,683 (3.91%)	
2008	47,870 (10.27%)	6,403 (9.03%)	4,606 (6.71%)	
2009	45,358 (9.73%)	5,851 (8.25%)	5,607 (8.16%)	
2010	49,894 (10.71%)	6,947 (9.79%)	6,881 (10.02%)	
2011	52,149 (11.19%)	7,982 (11.25%)	8,270 (12.04%)	
2012	50,235 (10.78%)	7,935 (11.18%)	9,050 (13.18%)	
2013	50,315 (10.80%)	8,755 (12.34%)	9,870 (14.37%)	
2014	50,620 (10.86%)	9,820 (13.84%)	10,745 (15.64%)	
2015	37,900 (8.13%)	7,835 (11.04%)	8,635 (12.57%)	
Bed size of hospital				
Small	81,509 (17.49%)	12,325 (17.37%)	11,853 (17.26%)	0.7656
Medium	119,456 (25.64%)	18,901 (26.64%)	18,066 (26.30%)	
Large	262,634 (56.36%)	39,334 (55.44%)	38,420 (55.94%)	
Unknown	2,369 (0.51%)	392 (0.55%)	344 (0.50%)	
Location/teaching status of hospital				
Rural	42,418 (9.10%)	5,068 (7.14%)	5,766 (8.40%)	<0.0001
Urban nonteaching	183,223 (39.32%)	25,069 (35.33%)	24,219 (35.26%)	
Urban teaching	237,958 (51.07%)	40,424 (56.97%)	38,354 (55.84%)	
Unknown	2,369 (0.51%)	392 (0.55%)	344 (0.50%)	
Region of hospital				
Northeast	80,848 (17.35%)	11,747 (16.56%)	12,061 (17.56%)	<0.0001
Midwest	117,620 (25.24%)	20,219 (28.50%)	21,013 (30.60%)	
South	177,236 (38.04%)	24,679 (34.78%)	25,050 (36.47%)	
West	90,263 (19.37%)	14,308 (20.17%)	10,558 (15.37%)	

Morbidly obese patients were noted to have a statistically significantly higher prevalence of deficiency anemia, congestive heart failure, chronic pulmonary disease, depression, uncomplicated and complicated diabetes, hypertension, hypothyroidism, liver disease, fluid and electrolytes disorders, psychoses, pulmonary circulation disorders, and renal failure in comparison with obese patients, who subsequently had a similar higher prevalence in these comorbidities in comparison with control subjects. A comparative list of Elixhauser comorbidity profiles is provided in Table 2.

**Table 2:** Elixhauser Comorbidity Profiles

Factor	Not Obese (n = 465,968)	Obese (n = 70,953)	Morbidly Obese (n = 68,682)	*P*
Acquired immune deficiency syndrome (AIDS)	185 (0.04%)	<10 cases	<10 cases	0.0347
Alcohol abuse	5,610 (1.20%)	1,079 (1.52%)	626 (0.91%)	<0.0001
Deficiency anemias	73,030 (15.67%)	12,268 (17.29%)	12,635 (18.40%)	<0.0001
Rheumatoid arthritis/collagen vascular disease	29,431 (6.32%)	4,085 (5.76%)	4,099 (5.97%)	0.0215
Chronic blood loss anemias	84,20 (1.81%)	1,336 (1.88%)	1,310 (1.91%)	0.6664
Congestive heart failure	23,732 (5.09%)	4,210 (5.93%)	6,200 (9.03%)	<0.0001
Chronic pulmonary disease	77,083 (16.54%)	14,826 (20.90%)	18,103 (26.36%)	<0.0001
Coagulopathy	13,839 (2.97%)	2,004 (2.82%)	2,288 (3.33%)	0.0370
Depression	67,245 (14.43%)	14,417 (20.32%)	15,340 (22.34%)	<0.0001
Uncomplicated diabetes	92,345 (19.82%)	20,196 (28.46%)	24,141 (35.15%)	<0.0001
Complicated diabetes	11,439 (2.46%)	3,355 (4.73%)	4,417 (6.43%)	<0.0001
Drug abuse	4,719 (1.01%)	955 (1.35%)	815 (1.19%)	0.0013
Hypertension	303,063 (65.04%)	53,988 (76.09%)	53,775 (78.30%)	<0.0001
Hypothyroidism	69,510 (14.92%)	11,892 (16.76%)	12,767 (18.59%)	<0.0001
Liver disease	7,274 (1.56%)	1,551 (2.19%)	1,550 (2.26%)	<0.0001
Lymphoma	1,956 (0.42%)	243 (0.34%)	236 (0.34%)	0.2092
Fluid and electrolyte disorders	52,131 (11.19%)	8,828 (12.44%)	10,997 (16.01%)	<0.0001
Metastatic cancer	871 (0.19%)	53 (0.08%)	83 (0.12%)	0.0030
Other neurological disorders	23,901 (5.13%)	4,024 (5.67%)	4,157 (6.05%)	<0.0001
Paralysis	1,909 (0.41%)	279 (0.39%)	291 (0.42%)	0.9200
Peripheral vascular disorders	12,417 (2.67%)	2,341 (3.30%)	2,162 (3.15%)	<0.0001
Psychoses	13,002 (2.79%)	2,745 (3.87%)	3,442 (5.01%)	<0.0001
Pulmonary circulation disorders	6,222 (1.34%)	1,422 (2.00%)	1,936 (2.82%)	<0.0001
Renal failure	30,362 (6.52%)	5,858 (8.26%)	7,092 (10.33%)	<0.0001
Solid tumor without metastasis	2,376 (0.51%)	318 (0.45%)	269 (0.39%)	0.1288
Peptic ulcer disease excluding bleeding	94 (0.02%)	<10 cases	<10 cases	0.4117
Valvular disease	21,333 (4.58%)	3,254 (4.59%)	2,434 (3.54%)	<0.0001
Weight loss	6,132 (1.32%)	982 (1.38%)	1,122 (1.63%)	0.0159

### Type and Reason for Revision

Information related to the type of rTKA is tabulated in Table 3. Across all three groups, all implants were most commonly revised. However, a statistically significant difference was found in the proportion of each revision between groups across all revisions. Reasons for revision are listed in Table 4. A significant difference was noted in the rate of reason for revision between all groups. Most interestingly, infection was the main reason for revision among all groups, followed by mechanical loosening, with rates of infection being significantly higher in morbidly obese compared with that in obese group, which in turn was higher than that in the control group (30.82% versus 25.2% versus 24.62%, respectively, *P* < 0.0001).

**Table 3:** Type of Revision, Stratified by Morbidly Obese, Obese, and Not Obese

Factor	Not Obese (n = 465,968)	Obese (n = 70,953)	Morbidly Obese (n = 68,682)	*P*
All implants	173,500 (37.23%)	25,816 (36.38%)	22,884 (33.32%)	<0.0001
Arthrotomy for removal of prosthesis	61,474 (13.19%)	9,864 (13.90%)	11,459 (16.68%)	<0.0001
Other, not otherwise specified	27,883 (5.98%)	3,384 (4.77%)	3,217 (4.68%)	<0.0001
Tibial insert	73,073 (15.68%)	11,302 (15.93%)	11,788 (17.16%)	0.0003
Tibial implant	96,694 (20.75%)	15,839 (22.32%)	15,452 (22.50%)	<0.0001
Patellar	33,090 (7.10%)	4,560 (6.43%)	4,317 (6.29%)	0.0002
Femoral implant	70,509 (15.13%)	11,462 (16.15%)	10,472 (15.25%)	0.0122

**Table 4:** Reason for Revision, Stratified by Morbidly Obese, Obese, and Not Obese

Factor	Not Obese (n = 465,968)	Obese (n = 70,953)	Morbidly Obese (n = 68,682)	*P*
Infection	114,719 (24.62%)	17,877 (25.20%)	21,171 (30.82%)	<0.0001
Mechanical loosening	91,987 (19.74%)	15,072 (21.24%)	14,062 (20.47%)	0.0003
Other mechanical problems	69,630 (14.94%)	11,058 (15.58%)	10,148 (14.78%)	0.1538
Implant failure	24,765 (5.32%)	3,084 (4.35%)	2,392 (3.48%)	<0.0001
Dislocation/instability	35,731 (7.67%)	6,321 (8.91%)	6,184 (9.00%)	<0.0001
Other mechanical complications	17,148 (3.68%)	2,643 (3.73%)	1,750 (2.55%)	<0.0001
Bearing surface wear	17,341 (3.72%)	2,379 (3.35%)	1,805 (2.63%)	<0.0001
Periprosthetic osteolysis	12,483 (2.68%)	1,845 (2.60%)	1,268 (1.85%)	<0.0001
Periprosthetic fracture	7,388 (1.59%)	937 (1.32%)	1,063 (1.55%)	0.0622

### In-hospital Complications and Resources Utilization

Morbidly obese patients were more likely to experience any complication than obese patients, followed by control subjects (34.56% versus 30.97% for obese versus 29.00% for control subjects, *P* < 0.0001). Morbidly obese patients were more likely to endure hematoma/seroma compared with control subjects, followed by obese patients (2.04% versus 1.68% versus 1.39%, respectively; *P* = 0.003), in addition to wound dehiscence (2.75% versus 1.65% for obese patients versus 1.46% in control subjects; *P* < 0.0001), postoperative infection (1.15% versus 0.88% for obese patients versus 0.79% for control subjects; *P* = 0.0002), pulmonary embolism (0.65% versus 0.53% in obese patients versus 0.41% for control subjects, *P* = 0.001), and postoperative anemia (30.17% versus 27.32% for obese patients versus 25.23% for control subjects; *P* < 0.0001). Obese patients had a lower likelihood of PVD complications than morbidly obese patients, followed by control subjects (0.006% versus 0.08% in morbidly obese patients versus 0.15% in control subjects, *P* = 0.0077), and equal likelihood for developing GI complications as morbidly obese patients, with both being lower than the control subjects (0.12% versus 0.12% in morbidly obese patients versus 0.22% in control subjects, *P* = 0.003). The overall data set is summarized in Table 5.

**Table 5:** Inverse Probability of Treatment Weighting Outcomes Analysis of Morbidly Obese Versus Obese Versus Not Obese Groups

Factor	Not Obese	Obese	Morbidly Obese	*P*
Any complication	29.00%	30.97%	34.56%	<0.0001
Central nervous system (CNS) complication	0.07%	0.07%	0.07%	0.9651
Cardiac complication	0.59%	0.62%	0.52%	0.5517
Peripheral vascular disease (PVD) complication	0.15%	0.06%	0.08%	0.0077
Respiratory complication	0.34%	0.33%	0.40%	0.5552
Gastrointestinal (GI) complication	0.22%	0.12%	0.12%	0.0030
Genitourinary (GU) complication	0.40%	0.30%	0.45%	0.2787
Hematoma/seroma	1.68%	1.39%	2.04%	0.0030
Wound dehiscence	1.46%	1.65%	2.75%	<0.0001
Postoperative infection	0.79%	0.88%	1.15%	0.0002
Deep vein thrombosis (DVT)	0.61%	0.65%	0.67%	0.6541
Pulmonary embolism (PE)	0.41%	0.53%	0.65%	0.001
Postoperative anemia	25.23%	27.32%	30.17%	<0.0001
Died during hospitalization	0.27%	0.18%	0.23%	0.1437
Length of stay (LOS) (d)	4.49	4.52	5.39	<0.0001
Total charges ($)	$72,299	$74,722	$83,083	<0.0001

Morbidly obese patients had a significantly greater average length of stay (LOS) (5.39 days) than obese patients (4.52 days) and control subjects (4.49 days; *P* < 0.0001). Morbidly obese patients also had significantly higher total charges ($83,083) than obese patients ($74,722) and control subjects ($72,299; *P* < 0.0001). Finally, no significant difference was observed in frequency of death during hospitalization between the control, obese, and morbidly obese groups (0.27% versus 0.18% versus 0.23%, respectively; *P* = 0.1437).

## Discussion

Obesity is a public health epidemic that is projected to encompass nearly half of the adult population in the near future.^23^ As a risk factor for the development of osteoarthritis, obesity has contributed to the increasing demand for primary TKA and is well known to be an independent risk factor for poor outcomes and increased complications after primary TKA.^24,25^ By contrast, a relative paucity of information exists on the demographic and comorbidity profile of obese patients undergoing rTKA and a lack of evidence on the outcomes among this patient population in the immediate in-hospital and postoperative period after rTKA. As such, this study sought to understand the epidemiologic characteristic of a growing population and investigate postoperative outcomes using a large national database because these data are critical for perioperative optimization and monitoring and patient counseling and education. Our analysis demonstrated that morbidly obese patients are at increased risk for worse complications and economic outcomes in the immediate postoperative period after rTKA compared with obese patients and that obese patients are at a greater risk than nonobese patients.

As expected, this study revealed a steadily growing rate of obesity and morbid obesity among rTKA recipients during the study duration, mirroring the general increasing rates of obesity among the US population. Morbidly obese group, more so than obese group, was noted to have a generally higher rate of young age, female patients, Black race, and Medicaid and private insurers as primary payors, in comparison with the control group. Both morbidly obese and obese patients have a higher prevalence of deficiency anemia, congestive heart failure, chronic pulmonary disease, depression, uncomplicated and complicated diabetes, hypertension, hypothyroidism, liver disease, fluid and electrolytes disorders, psychoses, pulmonary circulation disorders, and renal failure in comparison with control subjects, with a more pronounced prevalence among morbidly obese patients in comparison with obese patients. These epidemiologic and comorbidity profile findings are critical to understand and take into consideration whether efforts to design perioperative optimization protocols and pathways are to be undertaken.

This study highlighted the general trends in type and reason for rTKA at the national level and over an extended duration. In line with previously reported literature, infection and implant loosening remain the most common reasons for rTKA, with sequentially higher risk for morbidly obese patients for periprosthetic infections compared with obese patients and the latter compared with not obese patients.

Our analysis demonstrated increased risks of any in-hospital complication in both obese and morbidly obese groups when compared with the control cohort, and the morbidly obese group was at an even greater risk of complications than the obese group. Analysis of individual complications may help explain these differences. Compared With both control and obese groups, morbidly obese patients experienced hematoma/seroma, postoperative infection, wound dehiscence, and postoperative anemia at greater rates, followed by obese patients and then control subjects. Moreover, not obese patients had higher rates of hematoma/seroma when compared with obese patients. Morbid obesity acts as a technical challenge in revision arthroplasty and can lead to increased blood loss and dead space, which may allow for fluid collection and hematoma/seroma formation.^10,26^ These practical challenges are likely to also contribute to the markedly increased rates of postoperative infection and anemia, which this study demonstrated in morbidly obese patients. Furthermore, obesity is correlated with poor wound healing because of increased oxidative stress, decreased vascularity of adipose tissue, and molecular and cellular derangements that are present in obese individuals.^27^ In addition, obese patients are theorized to have paradoxical malnutrition, which further increases risk of wound infections.^27,28^ These concepts help explain the increased rates of postoperative infection and wound dehiscence that we observed in morbidly obese and obese patients. By contrast, obese patients were not at increased risk of seroma/hematoma formation when compared with the control subjects, suggesting a threshold of increasing BMI where the occurrence of these complications becomes significantly more likely.

Similarly, although obese patients were generally at greater risk for any complication compared with control subjects, this was not true for all complications. When analyzing individual complications, only postoperative anemia was significantly higher in the obese cohort when compared with that in not obese group. Interestingly, control subjects had a significantly higher risk of PVD, GI, and hematoma/seroma complications when compared with obese patients. Although counterintuitive, the paradoxical potentially protective effect of obesity on certain outcomes has been demonstrated in recent medical and surgical literature.^29,30^ Such studies empirically demonstrate a reverse J-shaped relationship between BMI and certain postoperative outcomes, with the highest rates of complications in morbidly obese and underweight patients, and the lowest rates in overweight and moderately obese patients. Several explanations have been proposed for these empirical findings, including increased lean body mass, protective peripheral body fat, reduced inflammatory response, genetics, and a decline in cardiovascular disease risk factors, although other unknown factors are likely to play a role.^30^ Overall, these factors suggest that overweight or moderately obese BMIs may be associated with better physiologic reserve, which may be protective against short-term outcomes after the surgical physiologic stress. These findings are not unprecedented in the arthroplasty literature and are in fact similar to a previous database study, which demonstrated increased rates of adverse events and minor complications in the morbidly obese compared with those in normal weight groups but not in the obese group compared with those in normal weight groups.^17^ Further studies are needed to identify the truly optimal BMI for revision arthroplasty procedures with particular focus on the effect of specific postoperative complication outcomes.

Regarding resources utilization, this study showed a linear relationship between BMI and total costs and LOS. This positive relationship between BMI and LOS after surgery has been previously demonstrated.^31-33^ A simple explanation for this relationship is that an increase in any postoperative complication as BMI increases would cause delays in discharge and increases in charges associated with treatment of those complications. In addition, patients with higher BMIs require longer duration of anesthesia, operative time, and total time in the operating room while undergoing TKA,^34^ and these findings can be potentially extrapolated for rTKA. These increased physiological stressors, in addition to baseline deficiencies in functional status in obese patients, may cause delays in meeting medical and physical therapy readiness criteria for discharge. As such, clinicians should be aware of the potential for increased LOS in morbidly obese patients and plan perioperative management accordingly.

This study had limitations that are inherent to large database studies. Although large databases provide high volumes of data representative of a national scale, they are prone to missing and erroneous data.^35^ Despite this propensity for errors, comorbidity and complication data in administrative records have been previously validated as accurate.^36^ As such, large national databases are routinely used to report on complications and in-hospital outcomes.^37-39^ Another limitation to this study is the ability to only report complications that occurred in the in-hospital postoperative period, given the nature of the registry used. While long-term follow-up of complications and functional outcomes after rTKA is critical, understanding the immediate in-hospital medical and economic outcomes is also of considerable value, which can be used to inform preoperative discussions and help create perioperative management protocols and plans. Finally, because this study stratified patients into groups of obesity and morbid obesity, we were unable to determine a specific BMI threshold where complications increase. Future studies are needed to determine the ideal BMI for optimal outcomes after revision arthroplasty and where the J-shaped complication curve begins to shift.

Despite these limitations, this study had several important strengths in design and scientific contribution. To the authors' knowledge, this is the largest study of its kind to present the trends of rTKA among not obese, obese, and morbidly obese patients and demonstrate adverse postoperative complication and economic outcomes in morbidly obese patients in the critical in-hospital postoperative period after rTKA on a national level. This uniquely increased risk faced by morbidly obese patients when compared with obese and not obese patients will prove useful in framing patient discussions and provide value in designing perioperative management protocols for these patient subsets. Furthermore, the findings of this study were strengthened by its propensity weighting statistical methodology, which allowed for the control of a large number of confounding demographic and medical comorbidities.

In summary, while both obesity and morbid obesity are risk factors for increased rates of any complication in the in-hospital period after rTKA, morbid obesity is a greater risk factor for adverse complication, LOS, and resources utilization. Moreover, obesity acts as a potential protective factor for certain postoperative outcomes, confirming the results of previous studies that introduced the concept of the obesity paradox and a J-shaped complication outcomes curve based on BMI. As the global obesity epidemic continues to burgeon, understanding the risks associated with obesity and morbid obesity after revision arthroplasty procedures is important. Future attention should be directed to better understanding of and establishing preoperative targets and to improving perioperative management protocols to optimize outcomes in this rapidly growing population.
